# Preoperative progressive pneumoperitoneum for giant inguinal hernias

**DOI:** 10.4103/0256-4947.65268

**Published:** 2010

**Authors:** Turgut Piskin, Cemalettin Aydin, Bora Barut, Abuzer Dirican, Cuneyt Kayaalp

**Affiliations:** From the Turgut Ozal Medical Center, Inonu University Faculty of Medicine, Department of General Surgery, Malatya, Turkey

## Abstract

Reduction of giant hernia contents into the abdominal cavity may cause intraoperative and postoperative problems such as abdominal compartment syndrome. Preoperative progressive pneumoperitoneum expands the abdominal cavity, increases the patient’s tolerability to operation, and can diminish intraoperative and postoperative complications. Preoperative progressive pneumoperitoneum is recommended for giant ventral hernias, but rarely for giant inguinal hernias. We present two giant inguinal hernia patients who were prepared for hernia repair with preoperative progressive pneumoperitoneum and then treated successfully by graft hernioplasty. We observed that abdominal expansion correlated with the inflated volume and pressure during the first four days of pneumperitoneum. Although insufflated gas volume can be different among patients, we observed that the duration of insufflation may be the same for similar patients.

Surgery of giant abdominal hernias can present problems by forcing and replacing hernia content into the diminished abdominal cavity. If most organs in the hernia sac create a second abdominal cavity, this can result in abdominal compartment syndrome after reduction of the sac content. Various methods have been recommended to overcome this problem. A prosthetic mesh usually closes large abdominal wall defects without problems. However, meshes do not eliminate the risk of compartment syndrome in reduced size abdominal cavities with giant hernias. Other methods to prevent compartment syndrome are creating space for the hernia with tissue expanders,^1^ debulking of the hernia content such as by omentectomy (even intestinal resection)^2^ or abdominal wall component separation techniques.^3^ Preoperative progressive pneumoperitoneum (PPP) has been used to maintain adequate abdominal space and is usually recommended for giant ventral hernias, but rarely for giant inguinal hernias. 1–9 We present two inguinal hernia patients who had massive organs in the hernia sac. These patients were prepared for operation with PPP and afterwards treated by mesh repair.

## CASE 1

**Figure 1:**
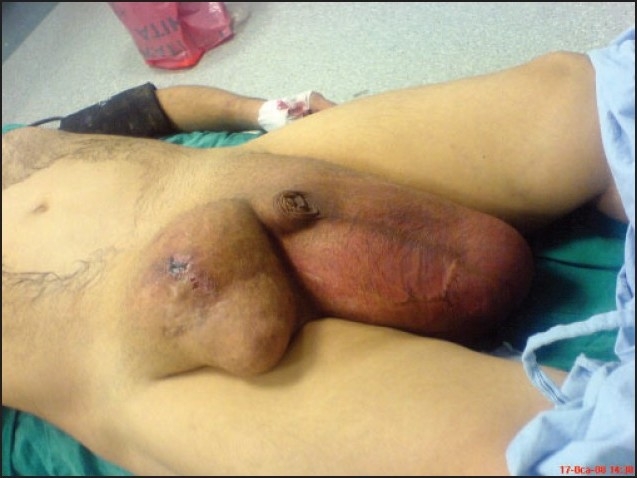
Giant bilateral inguinoscrotal hernia of the first patient.

**Figure 2:**
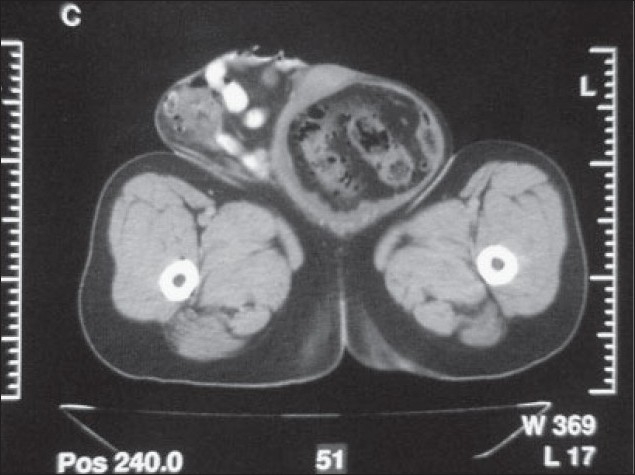
Intestinal segments at the level of femur on computed tomography.

**Figure 3:**
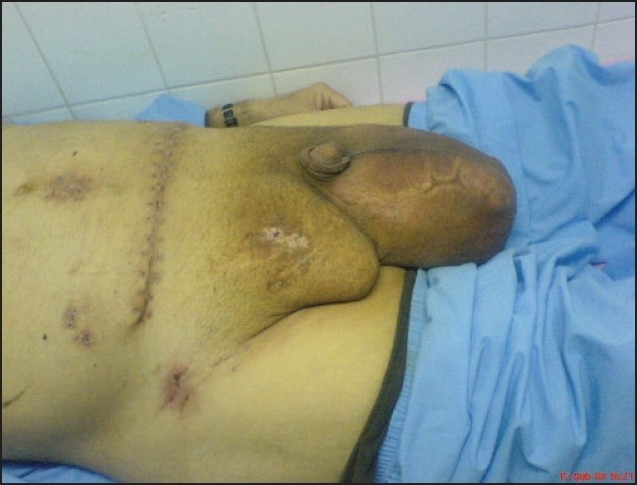
Postoperative view of the first patient.

A 56-year-old male presented to our clinic with bilateral giant inguinal hernias. His complaints were pain, bilateral inguinal mass and insufficient sexual activity. He was operated on four times before for right groin hernia. Concomitant diseases were arterial hypertension and diabetes mellitus. There were incision scars in the right groin region and bilateral giant inguinal hernias (Figure 1). Laboratory findings were normal except for a slightly elevated blood glucose. Contrast-enhanced CT revealed that omentum, small bowel and colon were in the bilateral hernia sacs (Figure 2). After evaluation of clinic signs and radiological findings, we decided to prepare the patient with PPP, after obtaining informed consent for the procedure. A peritoneal dialysis catheter was inserted into the abdominal cavity with a 3-cm lower midline incision under local anesthesia. Nitrous oxide was preferred for insufflation because of its inert structure. It was insufflated into the abdomen via a catheter. The first insufflation procedure was applied in the operating room and then at bed side (insufflator moved to the bed side). Daily insufflated gas volume, intraabdominal pressure and expansion of waist circumference at the level of umbilicus was recorded (Table 1). Insufflations went on for 18 days except day 11 because of a technical problem (Figure 3). We determined that abdominal expansion did not increase any more after day 13 despite increased intraabdominal pressure. Waist circumference at the level of the umbilicus expanded and stayed at 11 cm at that time. During the procedures, we gave third-generation quinolones to the patient and an analgesic, when necessary.

**Table 1:** Insufflated volume, intraabdominal pressure and expansion of the abdomen during preoperative progressive pneumoperitoneum of the patients.

Case 1			
Days	Nitrous oxide volume (cc)	Intra-abdominal pressure (mm Hg)	Expansion (cm)
1	2000	4	3.4
2	3100	5	6.5
3	4000	5	9.5
4	4500	8	11
5	5000	11	NA
6	5500	13	NA
7	5800	16	NA
8	6200	19	NA
9	6800	17	NA
10	6200	11	NA
11	NA	NA	NA
12	7100	21	NA
13	7000	21	21
14	7000	27	NA
15	7100	31	NA
16	7100	28	11
17	7200	28	NA
18	7400	29	NA

**Case 2**			
1	1500	8	2.5
2	2100	9	5
3	2500	13	10
4	3000	15	11

The patient was operated on under general anesthesia on day 19. A Pfannensteil incision was preferred and there was sigmoid colon and 500 cc of fluid in the left hernia sac. Some small bowel segments, appendix vermiformis, cecum, and 200 cc of fluid was in the right hernia sac. We performed appendectomy for prophylaxis. The hernia contents were dissected easily from the hernia sac and the bilateral hernia contents were pushed into the abdomen easily. The peritoneum was closed with absorbable sutures. Stoppa technique was preferred and a 30×30 polypropylene mesh was placed and fixed to bilateral spina iliaca anterior superior, symphysis pubis, Cooper ligaments, and midline. Suction drains were placed on the mesh and in the scrotum bilaterally. An abdominal incision was closed layer by layer and a peritoneal dialysis catheter was taken out. The scrotum was elevated postoperatively. Mild shortness of respiration and abdominal distention was seen on postoperative day one. On postoperative day two, recovery was excellent and the postoperative course was uneventful (Figure 3). Drains were removed on day 12 and the patient was discharged on postoperative day 14. A checkup after 11 months showed the hernia to be entirely cured.

## CASE 2

**Figure 4:**
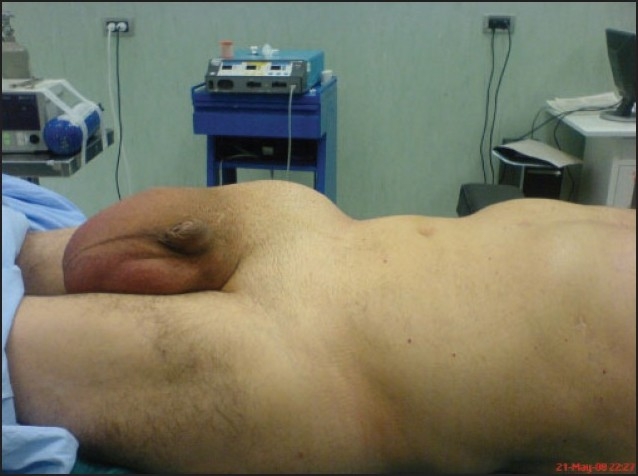
Preoperative picture of the second patient.

**Figure 5:**
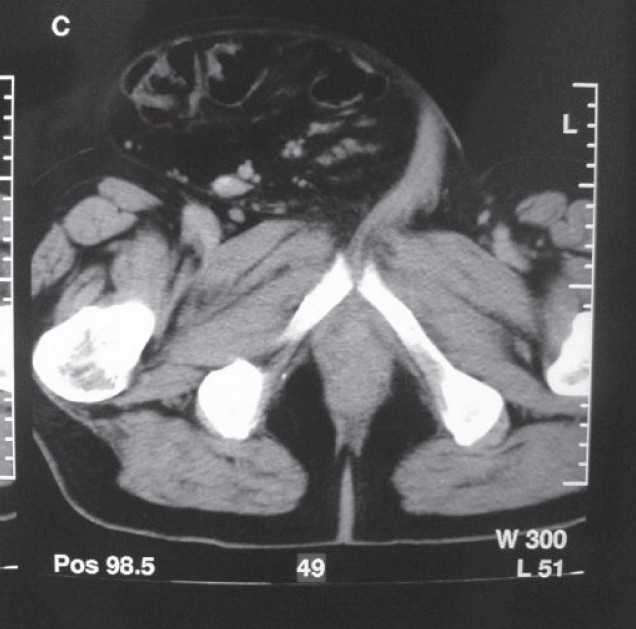
Preoperative computed tomography of the second patient.

A 63-year-old male presented with right giant inguinal hernia, pain and insufficient sexual activity. His concomitant diseases were coronary heart disease, arterial hypertension and diabetes. There was a giant right inguinal hernia and contrast-enhanced CT demonstrated omentum and a large mesenteric tissue with intestine segments in hernia sacs that displaced the penis laterally (Figure 4, 5). His laboratory findings were normal.

**Figure 6:**
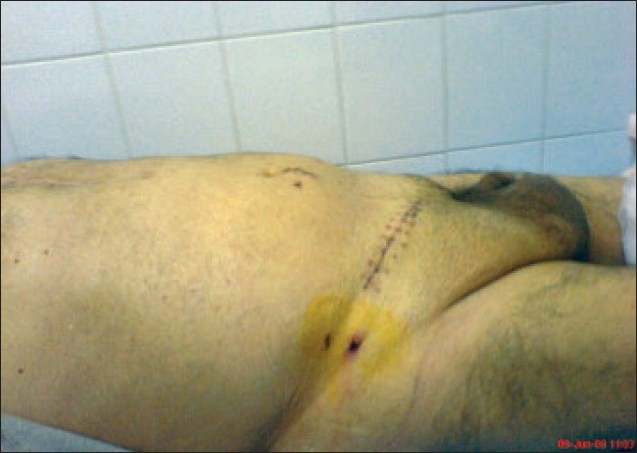
Postoperative picture of the second patient.

We decided that reduction of hernia sac contents could cause an increase in intraabdominal pressure postoperatively, so the patient was prepared for hernia repair with PPP after providing informed consent. A silicone Foley catheter was inserted into the abdominal cavity and insufflated as described in the first patient. The first pneumoperitoneum was applied in the operating room and then at the bedside (the insufflator was moved to the clinic from the operating room). Daily insufflated gas volume, intraabdominal pressure and expansion of waist circumference at the level of umbilicus was recorded (Table 1). The waist circumference at the level of the umbilicus increased to 11 cm on the fourth day. With the experience of the first patient, we decided that expansion was enough for operation and the patient was operated on on the fifth day. A standard inguinal incision was preferred under general anesthesia. Small bowel segments, appendix vermiformis, cecum, and 300 cc of free fluid were in the hernia sac. The hernia contents were pushed into the abdominal cavity easily and a zig maneuver performed after prophylactic appendectomy. The peritoneum was closed and a 7×15 cm polypropylene mesh was inserted and fixed to the symphysis pubis, inguinal ligament and conjoint tendon. A suction drain was placed on the mesh and in the scrotum. An abdominal silicone Foley catheter was taken out. The scrotum was elevated postoperatively. Mild difficulty on respiration was seen on the first postoperative day. The drains were removed on day 12 and he was discharged on postoperative day 13 (Figure 6). A checkup after 9 months was uneventful.

## DISCUSSION

Despite many reports of successful treatment of giant hernias by PPP, the procedure has not gained general acceptance. Introduction of alloplastic meshes promised the possibility of closing even large defects without problems.^5^ PPP was recommended for patients with giant hernias including a large amount of viscera in the hernia sac. These hernias are so-called “loss of domain” hernias because the contents of the hernia exceed the capacity of the abdominal cavity.^6^ Previously, there were two reported indications for PPP: when it would not otherwise be possible to perform the hernioplasty due to loss of domain and when forced reduction of the hernia sac might cause the patient to develop abdominal compartment syndrome postoperatively.^6^ We believe that liberal use of synthetic meshes diminished the need for PPP for the first indication, but PPP is still indicated in cases of a small abdominal cavity, which carries the risk of postoperative abdominal compartment syndrome.

We observed that hernia contents dissected from the sac were replaced into the abdomen without difficulty. It is reported that PPP increases the capacity of the retracted abdominal cavity, performs a pneumatic lysis of intestinal adhesions, allows the reduction of the hernia contents, and improves diaphragmatic function.^5^–^7^ PPP facilitates dissection of the hernial sac and locates other hernias or weak zones that are not evident in the initial examination. Stretching of the hernia sac has been found to be helpful in skin cleansing before the operation and potentially decreases the incidence of infections.^5^–^7^ PPP can be considered diagnostically as well as therapeutically and patients who cannot tolerate PPP should not be a candidate for any hernia repair treatment.^5^–^7^ PPP is contraindicated in cardiac and pulmonary insufficiency, and in patients with abdominal infections and incarcerated hernias.^2^,^5^ PPP has been used for different anatomic defects for ventral hernias, but was preferred for giant inguinal hernias less frequently. We reviewed articles on PPP in 130 patients, and found only seven patients with giant inguinal hernias.^2^–^8^ There were several catheter techniques in the literature such as central venous catheter, Foley or dialysis catheter.^2^–^9^ We first used a silicone dialysis catheter and later a silicone Foley catheter for the second case. Both were useful, but the Foley was cheaper.

Of the two types of gases available for pneumoperitoneum in our hospital, CO_2_ and NO, we preferred NO because it reabsorbs less rapidly than CO_2_. Other gases such as air can be used as well. In the literature there are no clear recommendations concerning the duration of PPP. PPP is maintained for between 7 and 60 days with a volume inflated between 1500 mL and 5000 mL. 4–9 We insufflated our first patient with 2000 to 7400 cc of gas during 18 days. The insufflated gas volume was dependent on patient tolerability. Intraabdominal pressure was monitored during the procedure. The maximum intraabdominal pressure was 29 mm Hg at day 18. Intraabdominal pressure was correlated with insufflated gas volume, but expansion of waist circumference at the level of umbilicus was not correlated with the insufflation volume and the pressure. Expansion length was 11 cm at day 16, the same as on day 4 (Table 1). We insufflated our second patient with 1500 to 3000 cc gas during four days. The maximum intraabdominal pressure was 15 mm Hg and the expansion length waist circumference at the level of umbilicus was 11 cm on the fourth day so we ended inflation after the experience with the first patient (Table 1). Expansions were enough for sufficient operation in both of our patients.

PPP is a safe and easy method for the prevention of postoperative abdominal compartment after repair of giant inguinal hernias. In our limited experience, we observed that abdominal expansion was correlated with the inflated volume and pressure only during the first four days. After four days, abdominal expansion was limited despite increased gas volume or pressure.
